# Survey of Individual and Institutional Risk Associated with the Use of Social Media

**DOI:** 10.5811/westjem.2016.2.28451

**Published:** 2016-05-05

**Authors:** Manish Garg, David A. Pearson, Michael C. Bond, Michael Runyon, M. Tyson Pillow, Laura Hopson, Robert R. Cooney, Jay Khadpe, Jason T. Nomura, Pholaphat C. Inboriboon

**Affiliations:** * Temple University Hospital, Department of Emergency Medicine, Philadelphia, Pennsylvania; † Carolinas Medical Center, Department of Emergency Medicine, Charlotte, North Carolina; ‡ University of Maryland School of Medicine, Department of Emergency Medicine, Baltimore, Maryland; § Baylor College of Medicine, Department of Emergency Medicine, Houston, Texas; ¶ University of Michigan, Department of Emergency Medicine, Ann Arbor, Michigan; || Conemaugh Memorial Medical Center, Department of Emergency Medicine, Johnstown, Pennsylvania; # SUNY Downstate Medical Center, Department of Emergency Medicine, Brooklyn, New York; ** Christiana Care Health System, Department of Emergency Medicine, Newark, Delaware; †† University of Missouri-Kansas City, Department of Emergency Medicine, Kansas City, Missouri

## Abstract

**Introduction:**

Residents and faculty in emergency medicine (EM) residency programs might be unaware of the professional and legal risks associated with the use of social media (SM). The objective of this study was to identify and characterize the types and reported incidence of unprofessional SM behavior by EM residents, faculty, and nurses and the concomitant personal and institutional risks.

**Methods:**

This multi-site study used an 18-question survey tool that was distributed electronically to the leaders of multiple EM residency programs, members of the Council of Emergency Medicine Residency Directors (CORD), and the residents of 14 EM programs during the study period May to June 2013.

**Results:**

We received 1,314 responses: 772 from residents and 542 from faculty. Both groups reported encountering high-risk-to-professionalism events (HRTPE) related to SM use by residents and non-resident providers (NRPs), i.e., faculty members and nurses. Residents reported posting of one of the following by a resident peer or nursing colleague: identifiable patient information (26%); or a radiograph, clinical picture or other image (52%). Residents reported posting of images of intoxicated colleagues (84%), inappropriate photographs (66%), and inappropriate posts (73%). Program directors (PDs) reported posting one of the following by NRPs and residents respectively: identifiable patient information (46% and 45%); a radiograph, clinical picture or other image (63% and 58%). PDs reported that NRPs and residents posted images of intoxicated colleagues (64% and 57%), inappropriate photographs (63% and 57%), or inappropriate posts (76% and 67%). The directors also reported that they were aware of or issued reprimands or terminations at least once a year (30% NRPs and 22% residents). Residents were more likely to post photos of their resident peers or nursing colleagues in an intoxicated state than were NRPs (p=0.0004). NRPs were more likely to post inappropriate content (p=0.04) and identifiable patient information (p=0.0004) than were residents.

**Conclusion:**

EM residents and faculty members cause and encounter HRTPE frequently while using SM; these events present significant risks to the individuals responsible and their associated institution. Awareness of these risks should prompt responsible SM use and consideration of CORD’s Social Media Task Force recommendations.

## INTRODUCTION

The use of social media (SM) continues to increase among both emergency medicine (EM) residents and attending physicians. In a recent investigation covering a one-year period, the number of EM and critical care blogs increased from 130 to 201 and EM, and critical care information dissemination increased 10% via Twitter^TM^ and 7% via Facebook^TM^.^1^ SM utilization in EM residency training programs is also on the rise. A recent survey of asynchronous education among 226 EM residents in 12 EM residency programs in the United States found that almost 70% of the residents endorsed EM podcasts as the most beneficial learning modality compared with textbooks, journals, and Google^TM^.^2^ In addition to asynchronous learning and educational resources, EM residency training programs use SM for professional marketing and for personal communication, socializing, and editorials.

The personal use of SM is of particular concern, as it could have substantial crossover professional implications. Healthcare workers in emergency departments (EDs) might share protected patient information on SM inappropriately or inadvertently, putting employees and institutions at risk. Furthermore, because the Accreditation Council for Graduate Medical Education (ACGME) has not yet established a standard for regulating the use of SM, EM residency programs have limited oversight of their departmental SM utilization. The combination of increased SM utilization, a lack of structured guidelines, and a lack of awareness of risk on the part of ED personnel presents substantial personal and institutional risk, as demonstrated by a number of recent highly publicized cases. For example, an EM physician was fired for a Facebook^TM^ public overshare, in which community members were able to identify a patient from a post.^3^

We thus sought to characterize observed SM behavior by EM residents, faculty, and nurses that carries potential personal and institutional risks. To our knowledge, this study by the Social Media Committee of the Council of Emergency Medicine Residency Directors (CORD) is the first investigation of its kind.

## METHODS

CORD is a national organization of faculty and program leaders for EM residencies accredited by the ACGME and the American Osteopathic Association. The CORD Social Media Committee developed an 18-question electronic survey tool (SurveyMonkey™, Palo Alto, CA), which was distributed in May 2013; reminders were sent two and four weeks later. The protocol and survey tool were approved by the institutional review board.

The electronic survey was distributed to the committee members’ home institutions, which created a diverse geographic sample of 432 residents. Additional residents were contacted, using the CORD listserv as a distribution method. The listserv consists of all CORD member program leaders, staff, and key faculty; these individuals were asked to distribute the electronic survey link to their programs’ residents. Faculty respondents consisted of the program directors (PDs), associate/assistant program directors, and core faculty members who were members of CORD and received the survey via the CORD listserv.

The survey tool is presented in Appendix A. Key measurements included the use of SM by residents, their knowledge of institutional policies regarding SM, and a comparison of SM use by residents and faculty members. Responses were voluntary and the study period was from May to June 2013. “Inappropriate” posts were left to the individual survey respondent to define, as a universal definition to cover everything is extremely difficult. We calculated descriptive statistics using the chi-squared test or Fisher’s exact test. StatsDirect software (v2.8.0, StatsDirect, Cheshire, UK) was used for all analyses.

## RESULTS

We received 1,314 responses: 772 from residents and 542 from faculty members. At the end of May 2013, the listserv had 841 faculty participants. According to the American Board of Emergency Medicine Report on residency training information for the academic year 2012 to 2013, there were 5,734 residents in accredited US categorical emergency medicine programs. Therefore, the survey response rate was 13% for residents and 44% for faculty. The participants’ demographics are summarized in Table 1. The faculty respondents’ geographic distribution was as follows: Northeast, 32%; South, 31%; Midwest, 27%; and West, 8%. The residents had a similar geographic distribution: Northeast, 33%; South, 33%; Midwest, 24%; and West, 8% (p=0.58).

Residents reported high-risk-to-professionalism events (HRTPE) resulting from their resident peers or nursing colleagues posting one of the following: identifiable patient information (177/680 [26%]); and a radiograph, clinical picture or other image (352/679 [52%]). Residents reported HRTPE created by residents and NRPs who posted images of intoxicated colleagues (564/674 [84%]), inappropriate photographs (445/675 [66%]), or inappropriate posts (490/672 [73%]). Some residents did not respond to all questions, which resulted in a lower denominator for some questions.

**Figure 1:**
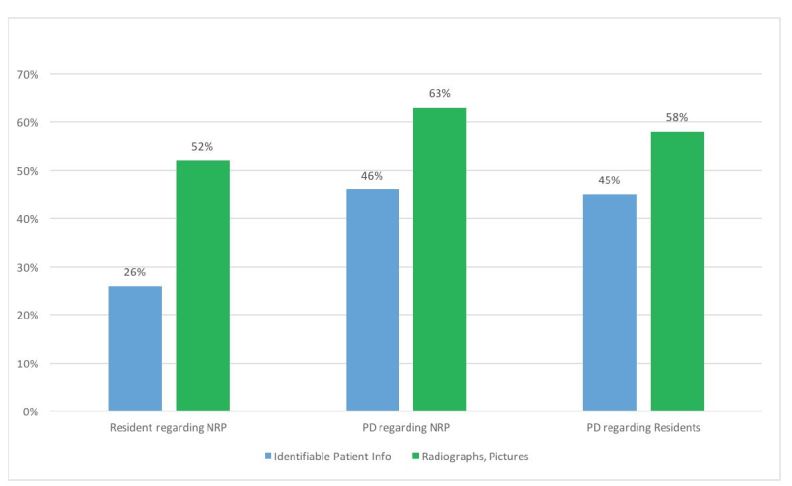
Reported high-risk-to-professionalism events by residents and program directors (PD) involving the posting of (1) identifiable patient information and (2) radiographs, clinical pictures or other images. Group 1=Resident responses regarding postings by non-resident providers (NRP). Group 2=PD responses regarding postings by NRPs (other faculty/nurses). Group 3=PD responses regarding postings by residents.

**Figure 2:**
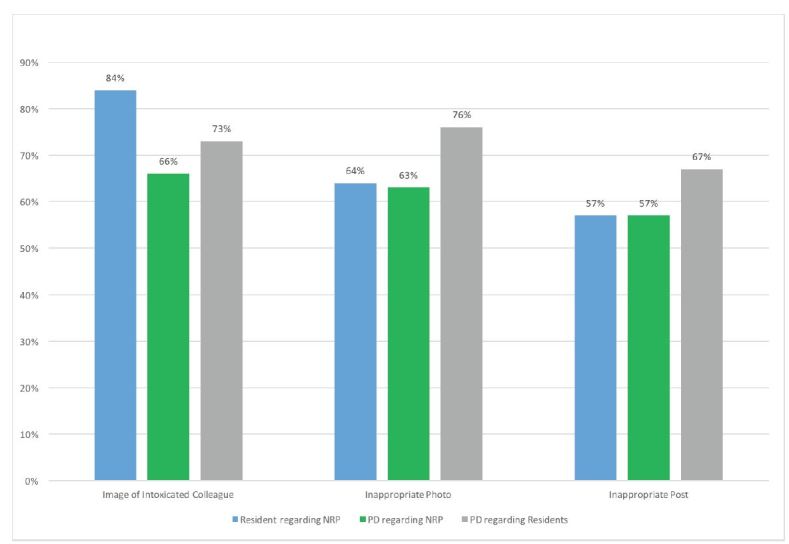
Reported high-risk-to-professionalism events by residents and program directors (PD) involving the posting of (1) images of intoxicated colleagues, (2) inappropriate photographs, or (3) inappropriate posts. *NRP*, non-resident providers

PDs reported HRTPE associated with non-resident providers (NRPs) and residents posting one of the following respectively: identifiable patient information (33/71 [46%] and 30/67 [45%]); and a radiograph, clinical picture or other image (44/70 [63%] and 39/67 [58%]) (Figure 1). PDs reported HRTPE related to posted images of intoxicated colleagues (44/69 [64%] and 38/67 [57%]); inappropriate photographs (45/71 [63%] and 38/67 [57%]); or inappropriate posts (54/71 [76%] and 44/66 [67%]) (Figure 2). PDs also reported being aware of or issuing reprimands or termination at least once a year (21/71 [30%] for NRPs and 15/67 [22%] for residents.

Residents were more likely to post photos of other residents or their nursing colleagues in an intoxicated state than were NRPs (p=0.0004). NRPs were more likely to post inappropriate content (p=0.04) and identifiable patient information (p=0.0004) than were residents. There was no difference for posting of inappropriate photographs (p=0.28) or radiographs, clinical pictures or other images (p=0.12) (Table 2).

## DISCUSSION

The practice of medicine mandates a high level of professionalism to ensure continued societal confidence in the profession and the physicians who deliver care. The EM Milestone project created by the ACGME includes two professionalism milestones. The first is “Professional Values,” which asks that the EM physician “demonstrates compassion, integrity, and respect for others as well as adherence to the ethical principles relevant to the practice of medicine.” The second is “Accountability,” which asks that the EM physician “demonstrates accountability to patients, society, profession and self.” The basic level of this second milestone requires that the EM resident “maintains patient confidentiality” and “uses social media ethically and responsibly.”^4^ Although these milestones are intended for the clinical care of patients, we believe it is equally important to ensure resident education regarding behavior outside the hospital setting. Due to the high number of inappropriate posts reported in this study, it is important that residents understand the potential pitfalls associated with the use of SM. Once SM posts are in the public sphere, it is often difficult, if not impossible, to remove them.

Institutional risk introduced by the personal use of SM has not been investigated in the literature. Half of the PDs who completed our survey reported being aware of at least one occurrence of the sharing of identifiable patient information by NRPs or residents during the last year. Interestingly, 25% of participating residents knew about similar postings by their peers and nurse colleagues of information that allowed a patient to be identified. Collectively, 207 survey respondents reported awareness of postings that allowed patients to be identified. It is impossible to know if these are distinct events, but it is clear that breaches of patient information on SM occur.

Confidentiality and privacy are fundamental expectations of the patient–physician relationship. The Health Insurance Portability and Accountability Act of 1996 (HIPAA) has privacy, security, and breach notification rules that are enforced by the Office for Civil Rights within the U.S. Department of Health and Human Services.^5^ Some of the observations and occurrences documented in our study fall within the scope of HIPAA and put individuals and institutions at risk.

In addition to federal ramifications for medical institutions in regard to unprofessional conduct on SM by employees, the individuals responsible for the HRTPE face state licensing consequences. In 2012, a national survey of state medical boards investigated physician violations of online professionalism and documented the subsequent disciplinary actions. For example, approximately 15% of state medical boards reported known physician violations resulting from online depiction of intoxication. Disciplinary consequences for all types of violations included letters of reprimand or probation; restriction, suspension or revocation of licensure; mandated education or community service; or monetary fines.^6^ Our results are more serious than those reported in this investigation. From the viewpoint of a residency PD, having nearly a quarter of the residents reporting seeing SM misuse should be cause for program-wide education and policy.

Consequences for individuals were reported by the participants in our study, but institutional consequences were never mentioned in the free-text responses. A few lawsuits have been filed against teaching institutions based on inappropriate SM utilization. The first is based on a posting, by a non-EM physician, of private information on Facebook^TM^ and Instagram^TM^ after evaluating an intoxicated female patient in the ED.^7^ The lawsuit names the physician, the hospital, and the affiliated medical school and is seeking $1.5 million in damages. The second suit occurred after non-physician hospital employees (allegedly including one nurse) posted a diagnosis of maternal syphilis on a Facebook^TM^ group with a large distribution.^8^ The lawsuit names the hospital employees and the medical center and is seeking more than $25,000 in damages. Though the number of serious violations is low, the incidence is likely to increase with larger payouts unless the responsible use of SM becomes a part of medical education either at the undergraduate or graduate level.

Regulation of the use of SM in residency training programs is hampered by the lack of clear guidelines from the accrediting body. The ACGME has not yet issued formal SM standards or guidelines for residency training programs, but the American Medical Association (AMA) has SM guidelines for physicians.^9^ Guidance tailored to the residency training environment is available from the CORD’s Social Media Task Force.^10^ The authors believe the AMA and CORD recommendations can help residency training programs use SM professionally and minimize personal and institutional risk. Professional use would include using SM for educational purposes, announcement of accolades and professional accomplishments while ensuring nothing unprofessional (e.g.: images of colleagues intoxicated, protected patient information, or posts that could be considered offensive by distinct groups) is posted.

Responsible SM use has many advantages for resident education. SM is interactive, brings together a geographically diverse distribution of information, and has near real-time peer review. If it is used unprofessionally or irresponsibly, SM can lead to serious personal, professional, and institutional consequences, including employment termination, fines, and federal violations. Our survey revealed violations of patient privacy and other inappropriate use of SM, and these represent risks to the residents and institutions that cannot be ignored as SM expands.

## LIMITATIONS

Our study has several limitations. First, the survey methodology could have led to inaccurate entry of the respondent data (i.e., if a respondent could have incorrectly selected a choice that did not reflect a true observation). Second, we asked respondents to self-report the frequency of observations, which is subject to recall bias (individuals who have knowledge of adverse events could be more likely to respond). Third, we left the definition of an inappropriate photograph up to the survey respondent. A finite definition was not provided knowing that individuals often make a value judgment of inappropriateness based on their own perceptions and life experiences. Finally, none of respondents actually reported any institutional consequence from the improper SM postings, despite the reports of individual consequences.

## CONCLUSION

Emergency medicine residents and faculty members who use SM frequently encounter HRTPE that hold potential personal and institutional risk. Awareness of these risks, and the risk of their own behaviors and potential liability should encourage responsible SM use and consideration of using the SM recommendations from the CORD’s Social Media Task Force.

## Supplementary Information



## Figures and Tables

**Table 1:** Demographic information for respondents (N=1314) in survey regarding inappropriate social media postings by emergency medicine residents and faculty.

Gender					Age (years)		
			
Male	Female	Prefer not to answer	Residents	Faculty (n=542)	<30	31–39	>40
63%	37%	1%	772	15% PDs; 85% other faculty	40%	39%	21%

*PDs,* program directors

**Table 2:** Comparison of reported high-risk-to-professionalism events (HRTPE) in program director (PD) responses regarding postings by other faculty and nurses versus resident responses regarding postings by other residents and nurses.

HRTPE	PD, non-resident, peers/colleagues	Resident, peers/colleagues	P-value
Intoxicated state	44/69, 64%	564/674, 84%	0.0004
Inappropriate content	54/71, 76%	490/672, 73%	0.04
Identifiable patient information	33/71, 46%	177/680, 26%	0.0004
Inappropriate photographs	45/71, 63%	445/675, 66%	0.28
Radiographs, clinical pictures or other images	44/70, 63%	352/679, 52%	0.12
