# Supplementary material for: Survey of Individual and Institutional Risk Associated with the Use of Social Media
Source: West J Emerg Med. 2016 May 5;17(3):344–9. doi: 10.5811/westjem.2016.2.28451 (PMC4899068; doi:10.5811/westjem.2016.2.28451)
Supplement: Supplementary file 1 [file wjem-17-344-s001.pdf]

Appendix A: Survey Tool

CORD Social Media Survey

1. What social networking sites do you currently use? Indicate all that apply
   - ☐ Facebook
   - ☐ Twitter
   - ☐ LinkedIn
   - ☐ YouTube
   - ☐ Ning
   - ☐ Blogs
   - ☐ None (I don't use social networking sites)
   - ☐ Other (please specify)

2. How do you utilize social networking sites for personal use? Indicate all that apply.
   - ☐ News
   - ☐ Entertainment
   - ☐ Videos
   - ☐ Research
   - ☐ Events
   - ☐ Networking (colleagues/peers)
   - ☐ Social (family/friends)
   - ☐ I don't use social networking sites

3. How often do you utilize social networking sites?
   - ☐ Multiple times a day
   - ☐ Daily
   - ☐ Several times a week
   - ☐ Weekly
   - ☐ Monthly
   - ☐ Infrequently enough to forget my password…

4. What is your level of interest in utilizing social media in the residency environment?
   - ☐ Very high
   - ☐ High
   - ☐ Neutral
   - ☐ Low
   - ☐ Very Low

5. Does your residency have an official social media policy?
   - ☐ Yes
   - ☐ No
   - ☐ In Process
   - ☐ Covered under Hospital, Corporate, or Institutional policy
   - ☐ Don't know

6. What barriers have you encountered in using social media? (Indicate all that apply)
   - ☐ Privacy Concerns

- ☐ Professional Boundary Concerns
- ☐ Sites Are Blocked
- ☐ Corporate Barriers
- ☐ Lack of Time/Too Busy
- ☐ Complicated/Confusing to Use

7. Please select the following items you would likely be interested in using social media to promote. Indicate all that apply.
   - ☐ Current Providers
   - ☐ New Providers
   - ☐ Open Positions/Hiring
   - ☐ Residency Program
   - ☐ Services – departmental services
   - ☐ Awards
   - ☐ Events -
   - ☐ Publications
   - ☐ Research
   - ☐ Articles / Journal Club
   - ☐ Videos
   - ☐ Other (please specify)

8. Does your residency have a social media page/site?
   - ☐ Yes
   - ☐ No
   - ☐ Not sure

9. If your residency has a social media page/site, who manages or acts as the administrator over the page/site?
   - ☐ Program Director
   - ☐ Associate or Assistant Program Director
   - ☐ Faculty Member (not PD or APD)
   - ☐ Program coordinator
   - ☐ Non-EM Med ED Coordinators/Administration
   - ☐ Resident
   - ☐ Other (please specify)

10. Please rate the following based on the scale: Strongly disagree, disagree, neutral, agree, strongly agree.
    - ☐ A faculty member and a resident should be able to be Facebook “friends”
    - ☐ A health care provider should be able to be Facebook “friends” with a patient if the patient offers
    - ☐ Social media interactions between faculty and residents have the potential to compromise evaluations during residency training
    - ☐ Social media interactions between faculty and residents have the potential for educational benefit
    - ☐ Social media interactions between health care providers and patients have the potential to compromise privacy
    - ☐ Postings on social media should be considered in resident evaluations of professionalism

- ☐ Postings on social media should be considered during evaluation of residency applicants

11. What is your age?
    - ☐ Age ≤ 30
    - ☐ Age 31-40
    - ☐ Age 41-50
    - ☐ Age 51-60
    - ☐ Age 60+

12. What is your gender?
    - ☐ Female
    - ☐ Male
    - ☐ Prefer not to answer

13. What region is your institution in?
    - ☐ Region 1 (Northeast): New England states: Maine, New Hampshire, Vermont, Massachusetts, Rhode Island, Connecticut; or, Mid- Atlantic states: New York, Pennsylvania, New Jersey
    - ☐ Region 2 (Midwest): East North Central states: Wisconsin, Michigan, Illinois, Indiana, Ohio; or, West North Central states: Missouri, North Dakota, South Dakota, Nebraska, Kansas, Minnesota, Iowa
    - ☐ Region 3 (South): South Atlantic states: Delaware, Maryland, District of Columbia, Virginia, West Virginia, North Carolina, South Carolina, Georgia, Florida ; or, East South Central states: Kentucky, Tennessee, Mississippi, Alabama, or West South Central states: Oklahoma, Texas, Arkansas, Louisiana
    - ☐ Region 4 (West): Mountain states: Idaho, Montana, Wyoming, Nevada, Utah, Colorado, Arizona, New Mexico ; or, Pacific states: Alaska, Washington, Oregon, California, Hawaii

14. What is your role in the program?
    - ☐ Resident **[Skip to Resident Section]**
    - ☐ Faculty

Faculty Section

15. How many years are you out of residency?
    - ☐ 0 to < 5 years
    - ☐ 5 years to < 10 years
    - ☐ 10 years or more

16. What is your current faculty position?
    - ☐ Program Director **[Skip to Program Director Section]**
    - ☐ Associate or Assistant Program Director **[End Survey]**
    - ☐ Core Faculty **[End Survey]**
    - ☐ Other Faculty Member **[End Survey]**

Program Director Section

17. As a residency program director, how often have you encountered the following from your peers/colleagues (include nursing and other faculty, exclude residents)? (Possible answers: Never, Once a Year, Multiple times ayear, Monthly, Weekly, Daily)
   - ☐ Posting involving identifiable patient Encounter
   - ☐ A posting involving deidentified patient encounter
   - ☐ Posting of a radiograph, clinical picture or other image
   - ☐ Posting of a peer/colleague in an intoxicated state
   - ☐ Posting resulting in one of your peers termination or reprimand
   - ☐ Inappropriate photographs of peers/colleagues
   - ☐ Inappropriate posts by peers/colleagues
   - ☐ Not applicable (not social media user)
   - ☐ Other (please explain in free text)

18. As a residency program director, how often have you encountered the following from your residents? (Possible answers: Never, Once a Year, Multiple times a year, Monthly, Weekly, Daily)
   - ☐ Posting involving identifiable patient encounter
   - ☐ A posting involving deidentified patient encounter
   - ☐ Posting of a radiograph, clinical picture or other image
   - ☐ Posting of a peer/colleague in an intoxicated state
   - ☐ Posting resulting in resident termination or reprimand
   - ☐ Inappropriate photographs of peers/colleagues
   - ☐ Inappropriate posts by peers/colleagues
   - ☐ Not applicable (not social media user)
   - ☐ Other (please specify)

19. Please add any additional experience (positive and negative) regarding social media and your institution: (Free Text)

[END Survey]

Resident Section

20. How often have you encountered the following from your peers/colleagues? (Skip if you do not utilize social media) [Possible answers: Never, Once a year, Multiple times per year, Monthly, Weekly, Daily]
   - ☐ Posting involving identifiable patient encounter
   - ☐ A posting involving deidentified patient encounter
   - ☐ Posting of a radiograph, clinical picture or other image
   - ☐ Posting of a peer/colleague in an intoxicated state
   - ☐ Inappropriate photographs of peers/colleagues
   - ☐ Inappropriate posts by peers/colleagues

21. Please add any additional experience (positive and negative) regarding social media and your institution: [Free Text]

22. What Post Graduate Level are you?
   - ☐ PGY-1
   - ☐ PGY-2
   - ☐ PGY-3

- ☐ PGY-4
- ☐ PGY-5

23. Which best describes the program in which you are currently enrolled?
    - ☐ Emergency Medicine
    - ☐ Emergency Medicine/Internal Medicine
    - ☐ Emergency Medicine/Pediatrics
    - ☐ Emergency Medicine/Family Medicine

24. Are you a chief resident?
    - ☐ Yes
    - ☐ No

Thank you for your participation.
